# Advanced hepatocellular carcinoma with hepatic vein tumor thrombosis and renal dysfunction after hepatic arterial infusion chemotherapy effectively treated by liver resection with active veno-venous bypass: report of a case

**DOI:** 10.1186/s12885-016-2749-4

**Published:** 2016-09-01

**Authors:** Atene Itoh, Hiroshi Sadamori, Kazuhisa Yabushita, Kazuteru Monden, Masashi Tatsukawa, Masayoshi Hioki, Tsuyoshi Hyodo, Kunihiro Omonishi, Toru Ueki, Satoshi Ohno, Kohsaku Sakaguchi, Norihisa Takakura

**Affiliations:** 1 Department of Gastroenterological Surgery, Fukuyama City Hospital, 5-23-1 Zao, Fukuyama, 721-8511 Japan; 2 Department of Internal Medicine, Fukuyama City Hospital, Fukuyama, Japan; 3 Department of Radiology, Fukuyama City Hospital, Fukuyama, Japan; 4 Department of Pathology, Fukuyama City Hospital, Fukuyama, Japan

**Keywords:** Hepatic arterial infusion chemotherapy, Hepatic vein tumor thrombosis, Hepatocellular carcinoma, Liver resection, Renal dysfunction, Veno-venous bypass

## Abstract

**Background:**

Hepatocellular carcinoma (HCC) patients with hepatic vein tumor thrombosis (HVTT) extending to the inferior vena cava (IVC) have an extremely poor prognosis. Here we report a case of HCC with HVTT and renal dysfunction after hepatic arterial infusion chemotherapy (HAIC) successfully treated by liver resection and active veno-venous bypass.

**Case presentation:**

A 77-year-old man was diagnosed to have a large HCC with intrahepatic metastases and HVTT extending to the IVC. Due to the advanced stage, HAIC with cisplatin was performed 13 times in a period of 17 months. As a consequence of this treatment, the size of the main HCC markedly decreased, and the advanced part of the HVTT went down to the root of the right hepatic vein (RHV). However, because of renal dysfunction, HAIC with cisplatin was discontinued and right hepatectomy with patch graft venoplasty of the root of the RHV was performed. Because progression of renal dysfunction had to be avoided, veno-venous bypass was activated during IVC clamping to prevent renal venous congestion and hypotension. Histological examination showed foci of a moderately differentiated HCC with extensive fibrosis and necrosis in the main HCC. Histologically, the HVTT in the RHV showed massive necrosis and tightly adhered to the vascular wall of the RHV. The postoperative function of the remnant liver was good, and no further deterioration of renal function was detected. The patient did not show signs of recurrence 15 month after surgery.

**Conclusion:**

In the present case, HAIC using cisplatin in combination with hepatic resection and patch graft venoplasty of the IVC provided a good long-term outcome with no HCC recurrence. Renal function was preserved by using active veno-venous bypass during IVC clamping to prevent renal venous congestion and hypotension.

## Background

Macrovascular invasion has been recognized as one of the most important prognostic parameters for patients with advanced hepatocellular carcinoma (HCC) [1, 2]. HCC patients with hepatic vein tumor thrombosis (HVTT) extending to the inferior vena cava (IVC) have an extremely poor prognosis [3, 4]. Surgical resection or chemotherapy can provide an acceptable long-term outcome in selected HCC patients with HVTT [5–7].

Here we report the case of a patient with advanced HCC showing HVTT extending to the IVC that was effectively treated by hepatic arterial infusion chemotherap**y** (HAIC) using powdered cisplatin (CDDP). Due to progressive renal dysfunction, HAIC was discontinued, and the liver was successfully resected with patch graft venoplasty of the root of the right hepatic vein (RHV). To avoid progression of renal dysfunction, active veno-venous bypass was used during IVC clamping, thus preventing renal venous congestion and hemodynamic instability.

## Case presentation

### Case report

A 77-year-old man was admitted to our hospital for the treatment of a liver tumor. His body mass index was 25 kg/m^2^, and he had a history of diabetes mellitus and hypertension. Laboratory tests on admission showed the following results: alanine aminotransferase (ALT), 68 IU/L (normal, 7–37 IU/L); aspartate amino transferase (AST), 104 IU/L (normal, 13–34 IU/L); serum albumin, 4.3 g/dL; prothrombin time/international normalized ratio (PT/INR), 0.99; total serum bilirubin, 0.8 mg/dL; and indocyanine green dye retention rate at 15 min (ICG-R15), 14.5 % (Table 1). The Child-Pugh score was 5; serum creatinine and estimated glomerular filtration rate (eGFR) were 1.25 mg/dL and 43.7 mL/min/1.73 m^2^, respectively. Serological findings for hepatitis B virus (HBV) and hepatitis C virus (HCV) were as follows: hepatitis B surface antigen (−), hepatitis B surface antibody (−), hepatitis B core antibody (−), and HCV antibody (−). Serum alpha-fetoprotein (AFP) was 46,300 ng/mL (normal, <10 ng/mL), and serum protein induced by vitamin K absence or antagonist (PIVKA-II) was 28,555 mAU/mL (normal, <28 mAU/mL).

**Table 1:** Laboratory data on admission

Complete blood count				HBV and HCV serology	
WBC	6,700/μL	ChE	292 IU/L	HBsAg	(-)
RBC	480 ×10^4^/μL	LDH	261 IU/L	HBsAb	(-)
Hb	14.1 g/dL	T-Chol	245 mg/dL	HBeAg	(-)
Hct	43.0 %	TP	7.4 g/dL	HBeAb	(-)
Plt	24.9 ×10^4^/μL	Alb	4.3 g/dL	HBcAb	(-)
		Na	139 mEq/L	HCVAb	(-)
Coagulation tests		K	4.8 mEq/L		
PT-INR	0.99	Cl	101 mEq/L	Tumor markers	
APTT	32.3 sec	Ca	9.4 mg/dL	AFP	46,300 ng/mL
Blood chemistry		UA	7.9 mg/dL	PIVKA-II	28,555mAU/mL
AST	104 IU/L	UN	17.6 mg/dL		
ALT	68 IU/L	Cr	1.25 mg/dL	Dye clearance test	
ALP	353 IU/L	CRP	0.28 mg/dL	ICG-R 15	14.5 %
γGTP	175 IU/L	HbA1c	7.5 %		
T.Bil	0.8 mg/dL	eGFR	43.7 mL/min/1.73 m^2^		

*AFP* alpha-fetoprotein, *Alb* albumin, *ALT* alanine aminotransferase, *ALP* alkaline phosphatase, *APTT* activated partial thromboplastin time, *AST* aspartate aminotransferase, *ChE* cholinesterase, *CRP* C-reactive protein, *eGFR* estimated glomerular filtration rate, *γGTP* gamma glutamyl transpeptidase, *HBV* hepatitis B virus, *Hb* hemoglobin, *HbA1c* hemoglobin A1c, *Hct* hematocrit, *HCV* hepatitis C virus, *ICG-R 15* indocyanine green dye retention rate at 15 min, *LDH* lactate dehydrogenase, *Plt* platelets, *PT-INR* prothrombin time-international normalized ratio, *RBC* red blood cells, *T.Bil* total bilirubin, *T.Chol* total cholesterol, *PIVKA-II* protein induced by vitamin K absence or antagonist, *TP* total protein, *UA* uric acid, *UN* urea nitrogen, *WBC* white blood cells

**Fig. 1:**
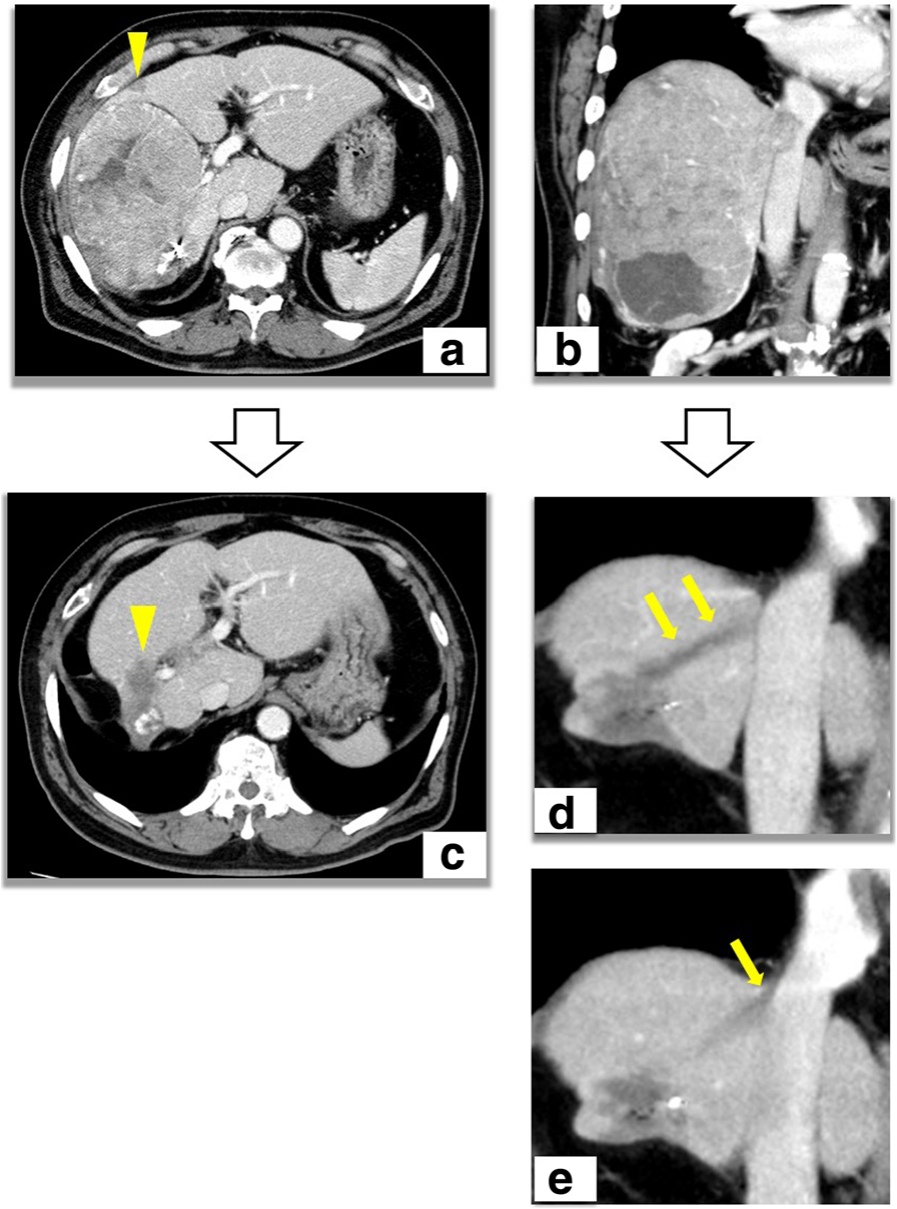
CT of the abdomen. **a** HCC with a maximum diameter of 16.0 cm in the right lobe detected before HAIC; intrahepatic metastasis is marked by the arrowhead. **b** HVTT extending from the RHV to the IVC prior to HAIC. **c** The size of the main HCC markedly decreased (*arrowhead*), with no obvious intrahepatic metastases after 13 courses of HAIC. **d**-**e** After 13 courses of HAIC, the advanced part of the HVTT went down to the root of the RHV (*arrows*)

Abdominal computed tomography (CT) showed a large HCC with intrahepatic metastasis (Fig. 1a) and HVTT extending from the RHV to the IVC (Fig. 1b). Because of the advanced stage of the HCC, HAIC was started by placing a standard angiography catheter in the right hepatic artery and subcutaneously connecting it to a port system (Piolax Medical Device Co., Ltd, Yokohama, Japan) inferior to the groin. Powdered CDDP, IA-call® (Nippon Kayaku Co., Ltd, Tokyo, Japan), was used for HAIC. CDDP was generally administered with a total dose of 65 mg/m^2^ via the right hepatic artery every 4–6 weeks. To prevent nephrotoxicity, adequate hydration was ensured before and after drug administration by intravenous infusion (1000–1500 mL of an infusion solution). After completing 11 courses of HAIC, serum creatinine increased to 1.8 mg/dL, and eGFR decreased to 29.2 mL/min/1.73 m^2^. Thus, the dose of CDDP was decreased by 50 % in the 12th and 13th courses of HAIC.

After 13 courses of HAIC, CT showed a marked decrease in the size of the main HCC, with no obvious intrahepatic metastases, thus indicating a partial response of the main HCC (Fig. 1c). Furthermore, the advanced part of the HVTT went down to the root of the RHV (Fig. 1d, e). Serum AFP and PIVKA-II levels decreased to 13 ng/mL and 15 mAU/mL, respectively (Table 2). However, after two months from the last HAIC, serum creatinine increased to 1.93 mg/dL, and eGFR decreased to 27.1 mL/min/1.73 m^2^. Because continuation of HAIC using CDDP became difficult due to progressive renal dysfunction, liver resection was selected as a radical treatment.

**Table 2:** Laboratory data after 13 courses of hepatic arterial infusion chemotherapy

Complete blood count				Tumor markers	
WBC	5,100/μL	ChE	307 IU/L	AFP	13 ng/mL
RBC	323 ×10^4^/μL	LDH	191 IU/L	PIVKA-II	15 mAU/mL
Hb	10.3 g/dL	T-Chol	245 mg/dL		
Hct	30.5 %	TP	7.0 g/dL	Dye clearance test	
Plt	14.6 ×10^4^/μL	Alb	4.5 g/dL	ICG-R 15	9.7 %
		Na	140 mEq/L		
Coagulation tests		K	5.4 mEq/L	CT volumetry	
PT-INR	1.11	Cl	106 mEq/L	Whole liver	1082 ml
APTT	28.0 sec	Ca	9.8 mg/dL	Right lobe	297 ml (27.4 %)
Blood chemistry		UA	8.6 mg/dL	Left lobe	785 ml (72.6 %)
AST	19 IU/L	UN	34.9 mg/dL		
ALT	14 IU/L	Cr	1.93 mg/dL		
ALP	231 IU/L	CRP	0.28 mg/dL		
γGTP	21 IU/L	HbA1c	5.8 %		
T.Bil	0.6 mg/dL	eGFR	27.1 mL/min/1.73 m^2^		

*AFP* alpha-fetoprotein, *Alb* albumin, *ALT* alanine aminotransferase, *ALP* alkaline phosphatase, *APTT* activated partial thromboplastin time, *AST* aspartate aminotransferase, *ChE* cholinesterase, *CRP* C-reactive protein, *eGFR* estimated glomerular filtration rate, *γGTP* gamma glutamyl transpeptidase, *HBV* hepatitis B virus, *Hb* hemoglobin, *HbA1c* hemoglobin A1c, *Hct* hematocrit, *HCV* hepatitis C virus, *ICG-R 15* indocyanine green dye retention rate at 15 min, *LDH* lactate dehydrogenase, *Plt* platelets, *PT-INR* prothrombin time-international normalized ratio, *RBC* red blood cells, *T.Bil* total bilirubin, *T.Chol* total cholesterol, *PIVKA-II* protein induced by vitamin K absence or antagonist, *TP* total protein, *UA* uric acid, *UN* urea nitrogen, *WBC* white blood cells

The results of liver function tests after 13 courses of HAIC were the following: ALT, 14 IU/L; AST, 19 IU/L; serum albumin, 4.5 g/dL; PT/INR, 1.11; total serum bilirubin, 0.6 mg/dL; and ICG-R15, 9.7 % (Table 2). The Child-Pugh score was 5. The volume of the whole liver was predicted to be 1082 mL by CT volumetry, and the volume of the estimated remnant liver (left lobe) was predicted to be 785 mL, resulting in an estimated resection rate of 27.4 %. Because of the 13 courses of HAIC, we assumed that the HVTT rigidly adhered to the wall of the RHV from the peripheral side to the root of the RHV, and a hepatectomy with patch graft venoplasty of the root of the RHV was planned. To avoid further progression of renal dysfunction, active veno-venous bypass was planned for preventing renal venous congestion and hypotension during IVC clamping.

**Fig. 2:**
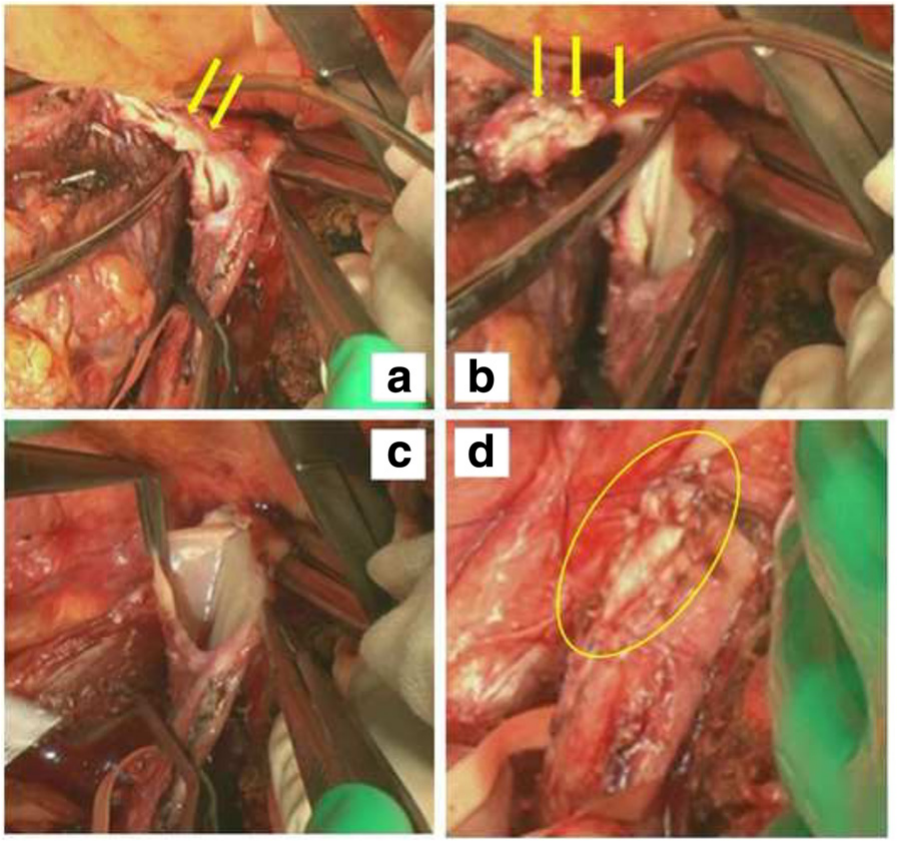
The maneuver during tumor thrombectomy and patch graft venoplasty. **a** Since the HVTT rigidly adhered to the wall of the root of the RHV (*arrows*), the root of the RHV was resected with the peripheral wall of the IVC. **b** As the HVTT had progressed to the cranial side and adhered to the wall of the IVC (arrows), the wall of the IVC at the cranial side of the root of the RHV was resected. **c** The surgical defect in the wall of the IVC measured 4.5 cm × 3.0 cm. **d** After the root of the RHV was resected with the peripheral wall of the IVC, patch graft venoplasty using proven bovine pericardial tissue (*circle*) was carried out for IVC reconstruction

After laparotomy via a thoracoabdominal incision, the right hepatic artery and the right portal vein were resected. Parenchymal transection for the right hepatectomy was performed by the anterior approach using the liver hanging maneuver. After the short hepatic veins were resected, the IVC was encircled at the suprahepatic IVC, the retrohepatic IVC just below the confluence of the common channel of the left and middle hepatic veins, and the retrohepatic IVC below the RHV. After cannulation of the axillary vein and the common iliac vein through the saphenous vein, veno-venous bypass using the Bio-Pump was activated. The IVC was clamped below the RHV with a DeBakey clamp and above the RHV with a straight vascular clamp, which was diagonally positioned to preserve the blood flow of the common channel of the left and middle hepatic veins. Since rigid adherence of the HVTT to the wall of the root of the RHV was suspected, the IVC wall located caudally to the root of the RHV was incised (Fig. 2a). The HVTT progressed to the cranial side and adhered to the IVC wall. Thus, the IVC wall at the cranial side of the root of the RHV was resected (Fig. 2b). The resultant surgical defect created in the wall of the IVC measured 4.5 cm × 3.0 cm (Fig. 2c). Reconstruction of the IVC was performed by patch graft venoplasty using bovine pericardial tissue (Edwards Lifesciences Co., Ltd, Tokyo, Japan), resulting in good patency of the IVC (Fig. 2d). IVC clamp time was 31 min, and the duration of active veno-venous bypass was 42 min. During IVC clamping, systolic blood pressure was maintained at around 90–110 mmHg.

**Fig. 3:**
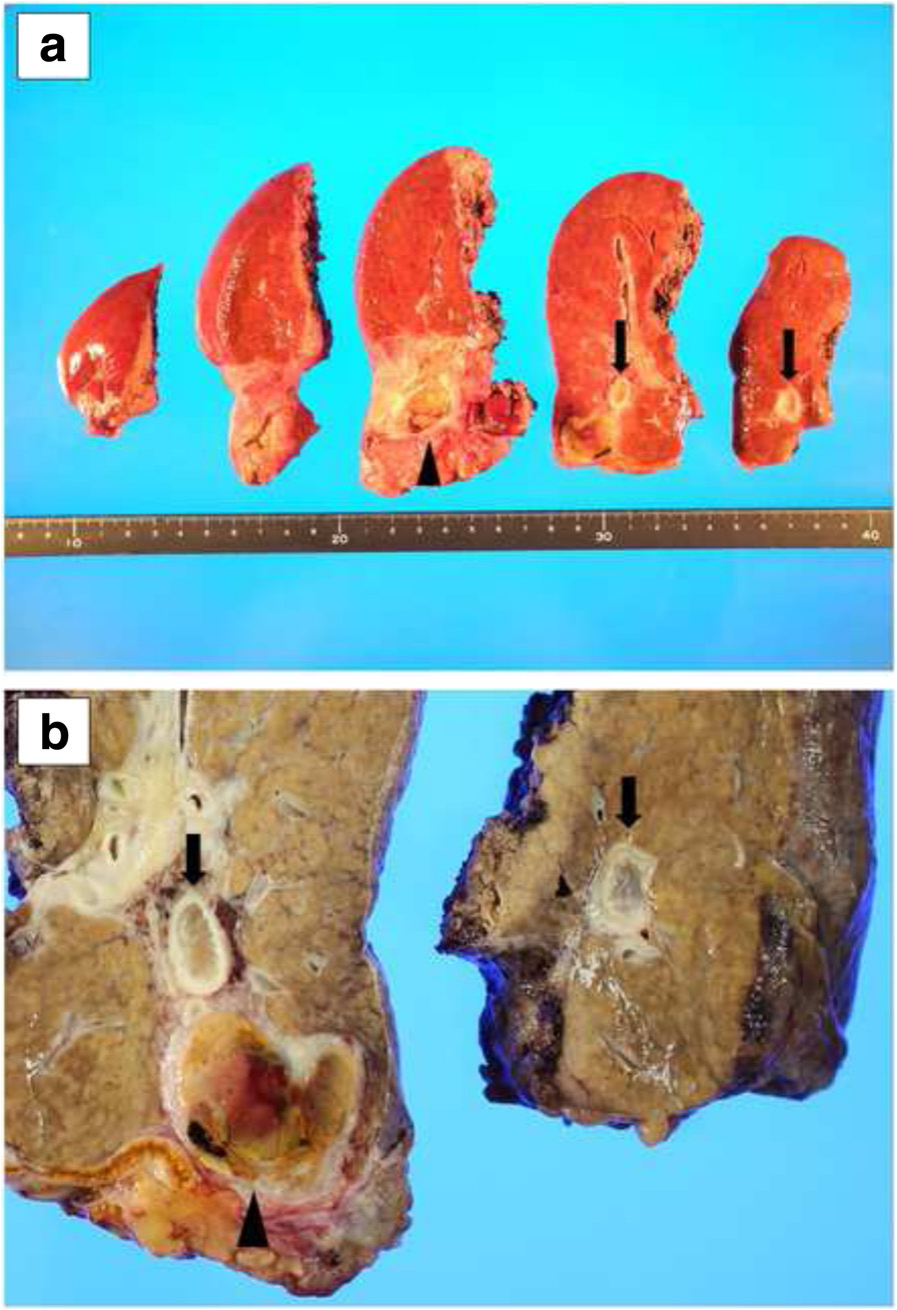
Macroscopic findings of the resected specimen. **a** The resected specimen before fixation. **b** The resected specimen after fixation. Macroscopic findings of the resected specimen showed the main HCC (*arrowhead*) and the HVTT adhering rigidly to the wall of the RHV from the peripheral side to the root of the RHV (*arrows*)

**Fig. 4:**
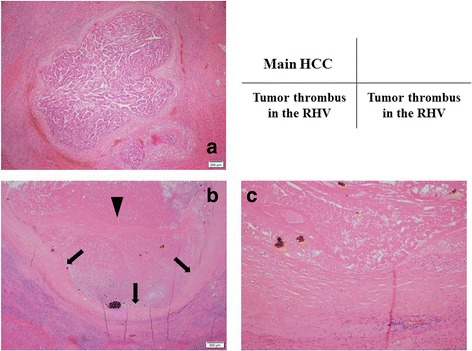
Microscopic findings of the resected specimen. **a** Foci of a moderately differentiated HCC were observed with extensive fibrosis and necrosis in the main HCC. **b** The HVTT in the RHV presented massive necrosis (*arrowhead*) and tightly adhered to the vascular wall of the RHV (*arrows*) in a wide area. **c** In the high-power field showing tight adhesion between the HVTT and the wall of the RHV, the endothelial cells of the RHV disappeared

Macroscopic findings of the resected specimen showed the main HCC and the HVTT that rigidly adhered to the wall of the RHV from the peripheral side to the root of the RHV (Fig. 3). Histological examination revealed foci of a moderately differentiated HCC with extensive fibrosis and necrosis in the main HCC (Fig. 4a). Histologically, the HVTT in the RHV showed massive necrosis and tightly adhered to the vascular wall of the RHV (Fig. 4b, c). The patient had an uneventful postoperative course, with good remnant liver function. Serum creatinine and eGFR were 1.52 mg/dL and 35.1 mL/min/1.73 m^2^, respectively, indicating preservation of renal function. The patient did not show signs of recurrence 15 months after the surgery.

## Discussion

Safety and efficacy of HAIC using powdered CDDP for advanced HCC have been demonstrated in several studies [8, 9]. Since Yoshikawa et al. [9] reported that the overall response rate was 33.8 % in 80 patients with advanced HCC without extrahepatic metastases, HAIC with powdered CDDP was selected to treat the advanced HCC presented in this report. By using this treatment, the size of the main HCC markedly decreased, and intrahepatic metastases became undetectable. Furthermore, the advanced part of the HVTT went down from the IVC to the root of the RHV, leading to patient survival with good quality of life for over 17 months. However, due to the progression of renal dysfunction, HAIC using CDDP could not be continued. Renal dysfunction has been already reported as a side effect of HAIC using CDDP in another study [9].

The antitumor activity of CDDP is cell cycle non-specific and mainly dependent on the concentration of non-protein-bound platinum (Pt) [10]. However, the blood concentration of non-protein-bound Pt markedly decreases after CDDP administration, and the half-life is less than 60 min. [11] Since arterial infusion of CDDP can provide a higher concentration of Pt in the tumor compared with intravenous infusion [12], HAIC using powdered CDDP is an excellent therapy in accordance with CDDP pharmacokinetics. In contrast, side effects of CDDP, including nephrotoxicity, are closely related to the blood concentration of non-protein-bound (Pt) [10]. Although CDDP-induced nephrotoxicity is transient and reversible in most cases, some of the patients with acute nephrotoxicity develop irreversible renal dysfunction [13]. In the present case, eGFR on admission was 43.7 mL/min/1.73 m^2^, indicating latent renal dysfunction due to diabetes mellitus and hypertension. It has been reported that nephrotoxicity increases with the dose and frequency of administration of CDDP and with the cumulative dose of CDDP [14, 15]. In the present case, the cumulative dose of CDDP reached 780 mg/m^2^ and may have caused irreversible renal dysfunction.

Usually HVTT extending to the IVC can be simply removed by thrombectomy without IVC wall resection, because the HVTT does not adhere to the wall of both the main hepatic vein and the IVC [16–18]. However, in the present case, we assumed that the HVTT rigidly adhered to the wall of the RHV from the peripheral side to the root of the RHV due to the 13 courses of HAIC. The preoperative CT scan showed suspected adhesions between the HVTT and the wall of the RHV and IVC, later confirmed by the macroscopic findings of the resected specimen. Thus, the root of the RHV was resected with the peripheral wall of the IVC, creating a surgical defect in the wall of the IVC measuring 4.5 cm × 3.0 cm. Although side clamping of the IVC might have been possible in this case, we decided to carry out total IVC clamping to achieve safety and certainty of the reconstruction of the surgical defect in the wall of the IVC. As a result, good patency of the IVC was achieved by patch graft venoplasty using proven bovine pericardial tissue, leading to the prevention of chronic renal venous congestion caused by IVC stenosis.

IVC thrombectomy is usually carried out under bleeding control by hepatic vascular exclusion (HVE) with or without extracorporeal bypass [16–18]. In the present case, the IVC could be clamped above the RHV for preserving blood flow to the common channel of the left and middle hepatic veins. Thus, HVE was unnecessary, and blood flow of the remnant liver was maintained during patch graft venoplasty of the IVC, preserving remnant liver function.

When IVC thrombectomy is simple and short, extracorporeal bypass during HVE might be unnecessary. However, when the duration of HVE is long enough to trigger hemodynamic instability, extracorporeal bypass from the IVC and/or portal vein to the superior vena cava (SVC) should be performed [18]. In the present case, since the HVTT adhered to the wall of the RHV and the IVC, resection of the root of the RHV including the peripheral wall of the IVC and patch graft venoplasty of the IVC were necessary. Thus, the duration of IVC clamping was longer than that of a usual IVC thrombectomy, and progression of renal dysfunction caused by the surgical procedure had to be avoided.

Venous congestion, rather than impairment of cardiac output, is associated with the development of acute kidney injury in acute heart failure [19, 20]. On the other hand, acute renal venous congestion has been closely related to acute renal failure in a clinical case of aortocaval fistula associated with ruptured aortic aneurysm and in an experimental model of aortocaval fistula that causes a rapid pronounced rise in central venous pressure [21–23]. Therefore, in the present case, veno-venous bypass from the IVC to the SVC using the Bio-Pump was activated to prevent renal venous congestion and hemodynamic instability during total IVC clamping. As a result, hemodynamic stability was maintained during IVC clamping, and postoperative renal function was preserved.

## Conclusions

Prognosis of HCC patients with HVTT extending to the IVC is poor [3, 4]. Surgical resection alone can provide long-term survival only in some HCC patients with HVTT [5, 6, 17]. Clinical trials of hepatic resection combined with chemotherapy, including HAIC, have been reported to improve the survival rate of HCC patients with macrovascular invasion [24–26].

In the present case, HAIC using CDDP in combination with hepatic resection and patch graft venoplasty of the IVC provided a good long-term outcome with no HCC recurrence. Furthermore, deterioration of renal dysfunction was avoided by using active veno-venous bypass during IVC clamping to prevent renal venous congestion and hypotension.

## Abbreviations

AFP: Alpha-fetoprotein
ALT: Alanine aminotransferase
AST: Aspartate amino transferase
CT: Computed tomography
eGFR: Estimated glomerular filtration rate
HAIC: Hepatic arterial infusion chemotherapy
HBV: Hepatitis B virus
HCC: Hepatocellular carcinoma
HCV: Hepatitis C virus
HVE: Hepatic vascular exclusion
HVTT: Hepatic vein tumor thrombosis
ICG-R15: Indocyanine green dye retention rate at 15 min
IVC: Inferior vena cava
PIVKA-II: Serum protein induced by vitamin K absence or antagonist
Pt: Platinum
PT-INR: Prothrombin time-international normalized ratio
RHV: Right hepatic vein
SVC: Superior vena cava
